# Trends in Fracture-Related Hospitalizations and Mortality in Brazil, 2015–2024

**DOI:** 10.3390/epidemiologia7030063

**Published:** 2026-05-04

**Authors:** Palloma Porto Almeida, Danielle Cabral Bonfim

**Affiliations:** Laboratory of Stem Cells and Bone Regeneration, Institute of Biomedical Sciences, Federal University of Rio de Janeiro, Rio de Janeiro 21941-902, Brazil; pahporto@gmail.com

**Keywords:** bone fractures, hospitalization trends, ethnic disparities, public health

## Abstract

Background/Objectives: Bone fractures represent a growing public health concern worldwide, yet national epidemiological assessments remain limited in Brazil. Understanding temporal trends, demographic disparities, and geographic heterogeneity is essential to guide prevention, resource allocation, and trauma-care planning. To characterize the epidemiological profile of bone-fracture-related hospitalizations and mortality in Brazil between 2015 and 2024, analyzing trends by sex, age, fracture type, and geographic and ethnic distribution. Methods: An epidemiological, observational, descriptive, and population-based ecological study was conducted using Hospital Information System of the Unified Health System SIH/SUS and IBGE data. Hospitalization rates, case fatality rates (CFR), relative risks, odds ratios, and Years of Life Lost (YLL) were calculated. Temporal trends were evaluated using Annual Percent Change (APC). Results: Other limb fractures were the most frequent injuries, while femur fractures showed the highest lethality. Men had nearly twice the hospitalization rate of women, driven by high-energy trauma in adults aged 20–59 years, whereas women experienced a sharp increase in femur-fracture admissions at older ages. Skull, facial, and thorax/pelvis fractures contributed disproportionately to premature mortality. Marked geographic and ethnic disparities were observed, with higher burdens in the North/Northeast and predominance among Brown and Indigenous populations. Conclusions: Fracture-related hospitalizations in Brazil have increased consistently, with distinct epidemiological patterns across demographic and regional groups. These findings highlight the need for targeted prevention and improved trauma-care strategies.

## 1. Introduction

Bone fractures represent a major public health concern worldwide and play an important role in morbidity across all age groups. From high-energy trauma in young adults to fragility fractures in older populations, these injuries are associated with substantial mortality, long-term disability, and significant reductions in quality of life [1,2]. Their impact extends beyond clinical complications, encompassing considerable direct costs related to hospitalization, surgical procedures, and rehabilitation, as well as indirect costs associated with loss of productivity, long-term caregiving demands, and reduced functional independence [3,4].

In Brazil, understanding the epidemiology of fractures is essential for guiding public health planning, improving resource allocation, and informing preventive strategies. The burden of these injuries varies according to demographic and social factors, including sex, age, and ethnic–racial background, mirroring global patterns of vulnerability [5]. Despite their relevance, systematized national data on hospitalization profiles, fracture-related mortality, and economic impact remain limited.

Although previous Brazilian studies have described fracture patterns, most have been restricted to specific fracture types, particularly femur fractures [6,7,8,9], or limited to regional analyses and elderly populations [10,11,12]. Comprehensive nationwide evaluations integrating multiple fracture categories, temporal trends, sex- and age-specific patterns, geographic variation, and ethnic distribution within a single analytical framework remain scarce. Furthermore, few studies have incorporated measures of premature mortality, such as Years of Life Lost (YLL), alongside hospitalization trends and case-fatality estimates. The availability of robust population-based databases, particularly the National Hospital Information System (SIH/SUS), provides a unique opportunity to address these gaps and generate a comprehensive national overview of fracture burden in Brazil.

Therefore, this study provides a nationwide and multi-dimensional epidemiological assessment of fracture-related hospitalizations and mortality in Brazil from 2015 to 2024. By integrating temporal trend analysis, sex- and age-specific stratification, fracture-type classification, geographic distribution, ethnic disparities, case fatality rates, and Years of Life Lost (YLL), this work aims to offer a comprehensive national overview of the fracture burden, addressing critical gaps in the Brazilian epidemiological literature.

## 2. Materials and Methods

### 2.1. Data Collection

This is an epidemiological, observational, descriptive, and population-based ecological study. The hospitalization and death data related to bone fractures were obtained from public domain sources, provided by the Hospital Information System of the Unified Health System (SIH/SUS) through the health information platform Tabnet via the electronic portal of the Department of Informatics of the Unified Health System (DATASUS). The dataset was extracted between 18 August and 21 August 2025, and stratified by sex, age, ethnicity, year, and state of hospitalization, referring to the period from 2015 to 2024.

Remaining life expectancy values data were obtained from the most recent period life tables (2024) published by the Brazilian Institute of Geography and Statistics (IBGE), stratified by sex and age. Annual estimates of the resident population by age group in Brazil were obtained from DATASUS/IBGE for the same period (2015–2024).

Fracture categories obtained from the SIH/SUS database are classified according to Chapter XIX of the International Classification of Diseases, 10th Revision (ICD-10) [13], which covers injuries to specific anatomical regions (codes S00–T98). In the database, fracture diagnoses were structured into anatomical groups based on ICD-10 codes. Accordingly, the following categories were analyzed: femur fractures (S72), including fractures of the femoral neck, intertrochanteric region, shaft, and distal femur; skull and facial bone fractures (S02), comprising fractures of the cranial vault, skull base, and facial bones; fractures of the neck, thorax, or pelvis, including cervical spine fractures (S12), rib, sternum, and thoracic spine fractures (S22), and lumbar spine and pelvic fractures (S32); fractures of other limb bones, including shoulder and upper arm fractures (S42), forearm fractures (S52), wrist and hand fractures (S62), lower leg fractures (S82), and foot fractures (S92), excluding femur fractures; and fractures involving multiple body regions (T02), corresponding to injuries affecting more than one anatomical region. These categories reflect the structural organization available in the national database and were maintained to ensure consistency and reproducibility of the analyses.

### 2.2. Calculation of Hospitalization Rates

Hospitalization rates per 100,000 inhabitants were calculated as follows:

$$Hospitalization\;rate\;\left(per\;100,000\right)=\;\frac{Total\;hospitalizations}{Mean\;population}\;\times \;100,000$$

This approach allowed for comparison of fracture burden between men and women, adjusted for population size. We report crude hospitalization rates per 100,000 inhabitants and age-stratified rates by predefined age groups; formal age-standardized rates using a reference population were not calculated.

### 2.3. Calculation of Years of Life Lost (YLL)

Data from SIH/SUS were displayed according to the Brazilian Ministry of Health categories: <1 year, 1–4 years, 5–9 years, 10–14 years, 15–19 years, 20–29 years, 30–39 years, 40–49 years, 50–59 years, 60–69 years, 70–79 years, and ≥80 years. Years of Life Lost (YLL) were calculated from aggregated mortality data stratified by sex and age group. Because individual ages at death were not available, each age group was assigned a representative age, corresponding to the midpoint of the interval. Remaining life expectancy was obtained from the 2024 Brazilian period life tables published by IBGE. A single reference life table was applied to all deaths occurring between 2015 and 2024 to ensure internal consistency and comparability across the study period. The open-ended category (≥80 years) was assigned a fixed representative age (89 years). For each age–sex stratum, total YLL was calculated as:

$$YLL_{\left(g\right)}=D_{\left(g\right)}\times \;{LE}_{\left(g\right)}$$

where *g* represents age–sex stratum and *D*_(*g*)_ represents the number of deaths, and *LE*_(g)_ the remaining life expectancy at the representative age of that stratum. The total YLL for each fracture category *c* and sex *s* was calculated as the sum over all deaths:

$${Total\;YLL}_{c}=\;\sum_{g\in c}{YLL}_{\left(c,g\right)}\;$$

The mean YLL per death was computed as:

$$Mean\;YLL_{c,s}=\frac{\;Total\;YLL_{c,s}}{Total\;Death_{s,c}}$$

Finally, the proportion of total YLL attributable to each fracture category was calculated as:

$${PropYLL}_{c,s\;}=\left(\frac{Total\;{YLL}_{C,S}}{\sum c\sum s{Total\;YLL}_{c,s}}\right)\times \;100$$

This approach provides both the absolute burden in years of life lost and the average YLL per death, allowing interpretation in terms of the typical years of life lost per fatal fracture, as well as the relative contribution of each fracture type to the overall YLL.

### 2.4. Case Fatality Rate (CFR)

Case Fatality Rates (CFRs) were calculated to estimate the lethality associated with each fracture type. CFRs (%) were calculated as the proportion of in-hospital deaths among all hospitalizations for each fracture category, sex, and year:

$$CFR=\;\frac{Deaths}{Hospitalization\;}\;\times \;100$$

To represent overall lethality by fracture type, data were aggregated across the entire study period (2015–2024), and an aggregate CFR (%) was computed for each fracture category as:

$$CFR=\left(\frac{\sum_{y}Deaths}{\sum Hospitalizations\;}\right)\times \;100$$

### 2.5. Statistics

Trends in fracture-related hospitalizations from 2015 to 2024 were analyzed using the Annual Percent Change (APC), estimated through a log-linear regression model. APC values and their 95% confidence intervals quantified the average yearly change in hospitalization rates. Age-stratified hospitalization rates per 100,000 inhabitants were calculated using the mean population of each age group.

The chi-square test of independence was applied to evaluate the association between sex and fracture type. Test assumptions were verified: no cell had an expected frequency < 1, and all cells had expected frequencies ≥ 5. Sex-specific odds ratios (ORs) with 95% confidence intervals were calculated for both hospitalization and mortality, allowing comparison of the relative risk of hospitalization and death between women and men for each fracture type.

To compare mean YLL between sexes for each fracture category, mean values were calculated at the age–sex stratum level and aggregated by fracture type. Comparisons between males and females were performed using a two-sample *t*-test, recognizing that YLL estimates were derived from midpoint-based approximations of aggregated mortality data.

Differences in the national distribution of fracture-related hospitalizations across ethnic groups were assessed using a chi-square goodness-of-fit test. When the overall test was significant, pairwise chi-square tests for equality of proportions were conducted, with Bonferroni correction applied for multiple comparisons. Given the nationwide scope and large sample size, statistical significance was interpreted in conjunction with effect size magnitude to avoid overemphasis on trivial differences. The analyses were based on predefined epidemiological outcomes and parameter estimates calculated separately for each fracture category. Except for ethnicity pairwise comparisons, which involved post hoc contrasts and were adjusted using Bonferroni correction, other analyses did not involve multiple simultaneous comparisons within a single inferential model and were therefore not subjected to global multiplicity correction.

In all equations, subscripts were used to denote specific dimensions of the dataset:

*c* represents the fracture category, according to ICD-10 classification;
*s* denotes sex (male or female);
*y* indicates the year of observation (from 2015 to 2024);
*g* refers to the age–sex stratum.

All statistical analyses were performed using RStudio software (version 2025.09.2); significance was defined as *p* < 0.05.

## 3. Results

### 3.1. Trends in Hospitalizations Between 2015 and 2024

**Figure 1:**
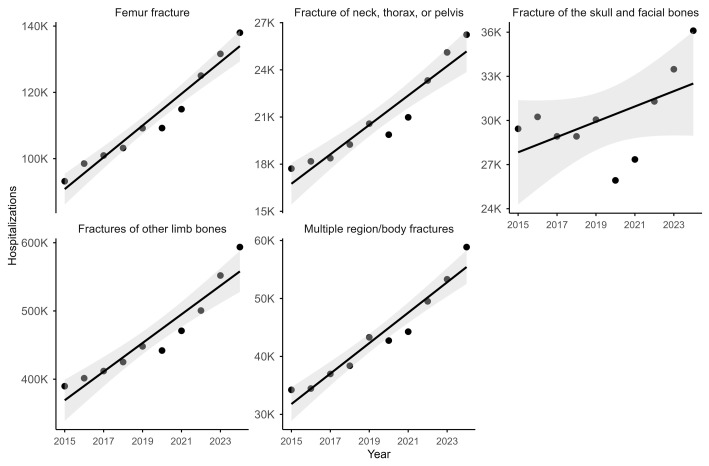
Annual trends in fracture-related hospitalizations in Brazil from 2015 to 2024, by fracture type. Data points represent observed annual hospitalization counts for each fracture category, and the solid line indicates the fitted linear trend with its 95% confidence interval, represented by the shaded gray areas. All values are shown on the original (non-logarithmic) scale.

The temporal analysis (2015–2024) revealed consistent upward trends in hospitalization rates across all fracture categories (Figure 1). Femur fractures showed a consistent linear increase over time, albeit at lower absolute volumes. In contrast, fractures of other limb bones exhibited both the highest baseline volume and the strongest linear growth, reinforcing their role as the primary contributor to fracture-related morbidity nationwide. A distinct deviation was observed for skull and facial fractures, which experienced a transient decline in 2020. A transient decline was observed in 2020. After 2021, the hospitalization rates for this fracture type resumed their upward trend, consistent with the patterns of the pre-pandemic years.

Analysis of hospitalization trends by fracture type, adjusted for population size, revealed variable annual percent changes (APCs) across fracture categories. Multiple region/body fractures exhibited the highest increasing over time, followed by fractures of other limb bones, while femur fractures and fractures of the neck, thorax, or pelvis showed comparable upward trends. For these categories, confidence intervals were narrow and entirely above zero (Table 1), indicating consistent and sustained growth throughout the study period. In contrast, skull and facial fractures demonstrated a smaller and non-significant change, with confidence intervals overlapping zero, suggesting relative temporal stability.

### 3.2. Fatality

The analysis of fracture-related case fatalities (CFR) reveals significant variation by fracture type. Femur fractures presented the highest fatality rate (3.1%), followed by fractures of the neck, thorax, or pelvis (2.6%), multiple region fractures (1.8%), skull and facial fractures (0.8%), and other limb fractures (<0.5%).

**Figure A1:**
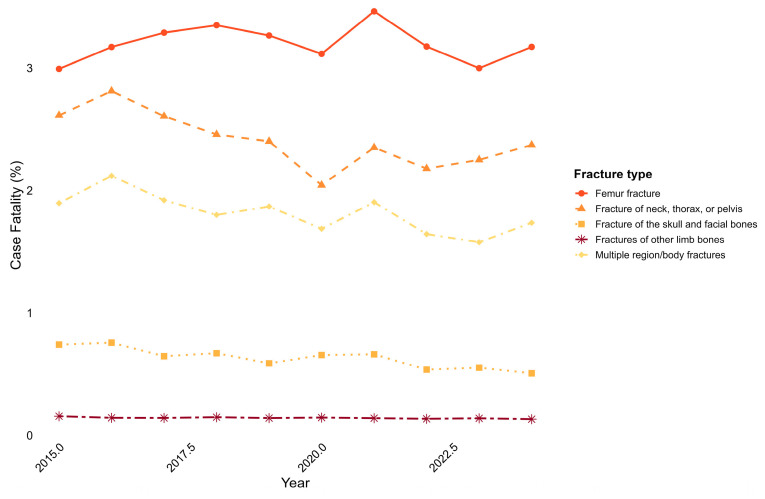
Annual case fatality rates (%) for fracture-related hospitalizations in Brazil, 2015–2024, by fracture type. The case fatality rate was defined as the percentage of in-hospital deaths among all hospitalizations for each fracture category. Values represent annual percentages based on SIH/SUS records.

Analysis of CF by fracture type between 2015 and 2024 revealed marked heterogeneity in severity. Femur fractures consistently exhibited the highest lethality, with annual CFRs ranging from 2.9% to 3.5%, and a notable peak in 2021 (3.46%). Fractures of the neck, thorax, or pelvis also showed elevated CFRs between 2.0% and 2.8%, with a decline in 2020 followed by an increase in subsequent years. Multiple region fractures maintained intermediate lethality, fluctuating between 1.6% and 2.1%, while skull and facial fractures remained below 0.8%, and fractures of other limb bones had the lowest CFR (<0.2%) (Figure A1). Overall, CFR trends remained relatively stable across the study period, with a transient deviation observed in 2020.

### 3.3. Sex Differences in Hospitalization Rates

In the analysis of hospitalizations among men and women from 2015 to 2024, adjusted for population size, men exhibited a higher cumulative rate of 4600 per 100,000 inhabitants, approximately twice that of females (2265 per 100,000 inhabitants), indicating a greater overall risk of fracture-related hospitalizations among men. A chi-squared test was applied to evaluate the association between sex and fracture type. The analysis revealed a highly significant result (χ^2^ = 57,092, df = 4, *p* < 0.001), indicating that the distribution of hospitalizations due to fractures is not independent of sex.

Women presented a lower absolute number of hospitalizations due to femur fractures compared with men. However, within the female fracture profile, femur fractures were the second leading cause of admission, accounting for 16.5% of female fracture-related hospitalizations. Women had higher odds of hospitalization due to femur fractures (OR 1.55; 95% CI 1.54–1.56), and femur fractures were associated with higher case fatality in females (6.86%) compared with males (2.83%). Fractures of other limb bones represented the major cause of hospitalizations in both sexes, with similar proportional contributions (71.6% in women and 73.4% in men); despite their high frequency, these fractures showed very low fatality rates (0.14–0.16%).

In contrast, sex-specific patterns became more pronounced for fractures associated with high-energy trauma: men displayed considerably higher odds of sustaining skull and facial fractures (OR 0.48; 95% CI 0.50–0.51) and fractures of the neck, thorax, or pelvis (OR 0.80; 95% CI 0.79–0.81). These injuries were markedly more frequent in males (256,554 skull/facial fractures vs. 56,994 in females), contributing to distinct sex-specific hospitalization patterns (Table 2).

**Figure 2:**
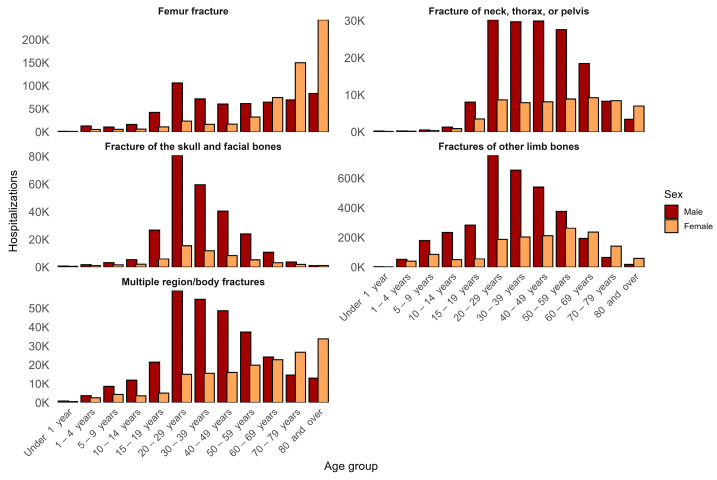
Distribution of fracture-related hospitalizations in Brazil from 2015 to 2024 by sex, age group, and fracture type. Stacked panel bar charts display the absolute number of hospitalizations for each fracture category across age groups, stratified by sex. Bars represent annual counts aggregated over the study period, allowing comparison of age- and sex-specific hospitalization patterns within each fracture type.

When analyzing hospitalizations by sex and age group, fractures of other limb bones accounted for the highest number of admissions among men, totaling 2 871 835 hospitalizations between 20 and 59 years of age. All fractures, except for femur fractures, followed the same pattern related to the number of admissions of the male gender. The age group with the greatest concentration of cases was 20–29 years, with 754,956 admissions, forming the first peak of a clear bimodal distribution in males, with a second increase after 50 years. In contrast, women exhibited a markedly different distribution: femur fractures were the leading cause of hospitalization in older adults, with 242,702 admissions in those aged 80 years or older, followed by fractures of other limb bones among women aged 60–69 years (236,501 hospitalizations) (Supplementary Data S1). Figure 2 illustrates the age- and sex-specific distribution of hospitalizations across fracture types. Skull, facial, and thorax/pelvis fractures were also more prominent among men aged 20–49 years, further reinforcing the sex-specific mechanisms underlying fracture risk (Figure 2).

### 3.4. Years of Life Lost by Fracture Type

Analysis of YLL due to fracture-related deaths showed differences by fracture type and sex. Femur fractures accounted for the largest share of total YLL, representing 25.8% in females and 22.3% in males, with mean years of life lost being 10 and 14 years, respectively, consistent with mortality occurring predominantly at older ages. In contrast, fractures of the neck, thorax, or pelvis contributed proportionally less to total YLL (2.97% in females and 11.3% in males), but exhibited higher mean YLL per death (23 and 27 years). Skull and facial fractures showed the smallest contribution to overall YLL (0.92% in females and 5.6% in males) yet had the highest mean YLL (32 and 34 years). Fractures of other limb bones accounted for 4.6% of total YLL in females and 13.4% in males, with mean YLL of 19 and 28 years, respectively. Multiple-region fractures contributed 5.3% and 7.8%, with 11 and 19 mean years lost, respectively. All sex comparisons in mean YLL were statistically significant (*p* < 0.001).

### 3.5. Geographical Distribution of Hospitalizations

**Figure A2:**
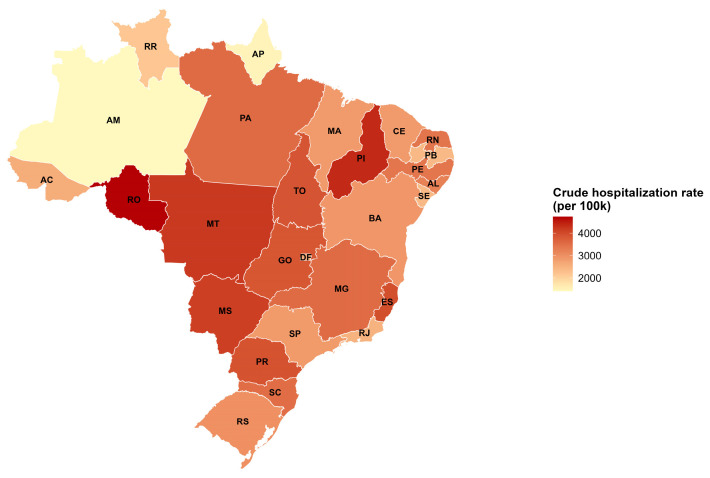
Population-adjusted hospitalization rates for bone fractures across Brazilian states, 2015–2024. Choropleth map showing hospitalization rates per 100,000 inhabitants for all fracture types combined. Rates were calculated using SIH/SUS hospitalization records and population estimates from IBGE. Colour intensity represents the magnitude of the rate in each state.

Population-adjusted hospitalization rates for bone fractures varied widely across Brazilian states (Figure A2). The highest rates were concentrated in Rondônia (4745 per 100,000), Piauí (4402), and Mato Grosso (4200), followed by intermediate values in the South and Southeast (2500–3500 per 100,000). In contrast, Amazonas and Acre recorded the lowest hospitalization rates, below 2000 per 100,000 inhabitants (Supplementary Data S2). Overall, the data reveal a spatially heterogeneous distribution of fracture-related hospitalizations.

### 3.6. Ethnic Distribution of Hospitalizations

**Figure A3:**
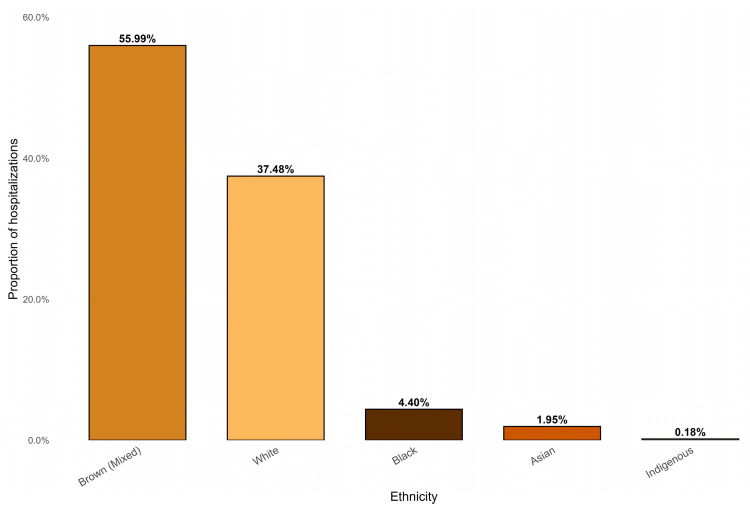
Proportion of fracture-related hospitalizations in Brazil, 2015–2024, by ethnicity. Proportions represent the distribution of SIH/SUS-recorded hospitalizations across ethnic groups. Pairwise differences between ethnic groups were assessed following a significant chi-square test, with *p*-values adjusted using the Bonferroni correction (*p* < 0.05).

Regarding the ethnicity profile of the hospitalized patients, the majority were Brown (Mixed), corresponding to 55.99% of all cases, followed by the White ethnicity (37.48%). All pairwise differences between ethnic groups were statistically significant (Bonferroni-adjusted *p* < 0.05), indicating that the distribution of fracture-related hospitalizations differs meaningfully across ethnic groups and that each ethnicity contributes a distinct proportion to the overall hospitalization burden (Figure A3).

**Figure 3:**
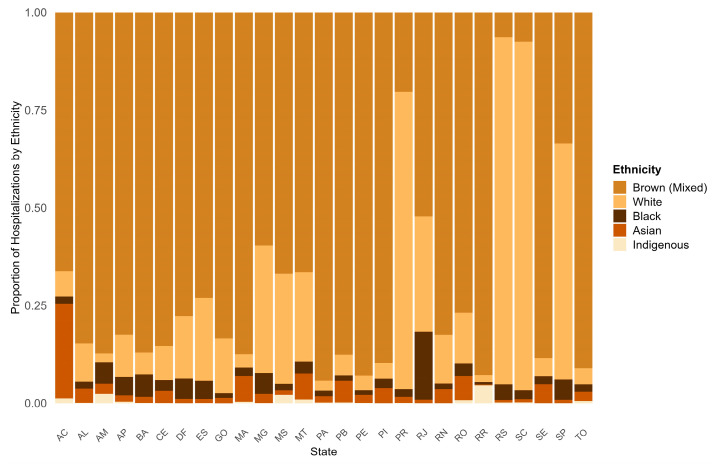
Distribution of fracture-related hospitalizations by ethnicity across Brazilian states, 2015–2024. Stacked bar chart displaying the proportional distribution of SIH/SUS-recorded hospitalizations for each ethnicity category within each Brazilian state.

Regarding the place of hospitalizations, the highest absolute numbers were observed in Minas Gerais for Asian and Brown (Mixed) individuals, and in São Paulo for Black and White individuals. Brown (Mixed) individuals were the predominant group among hospitalizations across most states, exceeding 50% in nearly all regions. Particularly high hospitalization rates were observed in states such as Pará (94.2%), Pernambuco (92.9%), Tocantins (91.1%), Piauí (89.7%), and Bahia (87.1%). The exceptions were Paraná, Rio Grande do Sul, Santa Catarina, and São Paulo, where White individuals were the majority (76.0%, 88.8%, 89.2%, and 60.4% of hospitalizations, respectively) (Figure 3).

**Figure 4:**
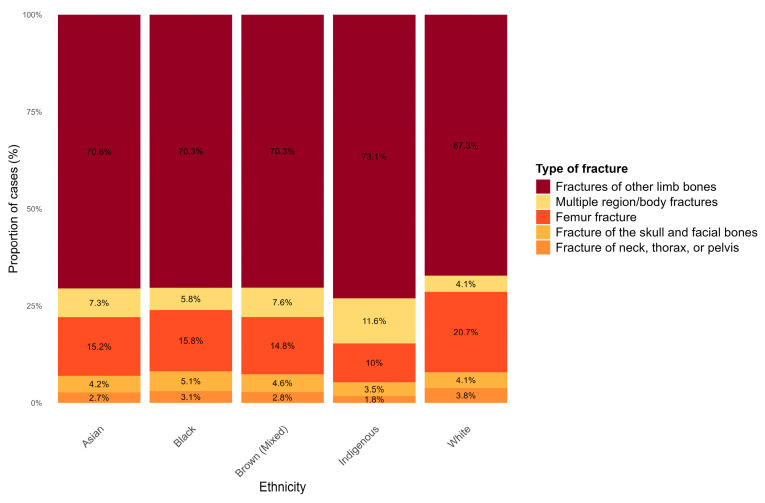
Relative distribution of fracture types across ethnic groups in Brazil, 2015–2024. Stacked bar chart showing the proportional distribution of SIH/SUS-recorded fracture-related hospitalizations within each ethnic group across five fracture categories.

Relative to fracture type across ethnicities, fractures of other limb bones accounted for the highest proportion overall, particularly among Indigenous individuals (73.1%), who also exhibited the highest proportion of hospitalizations due to multiple region/body fractures (11.6%). White individuals showed the highest proportion of femur fractures (20.6%), while Black individuals had the greatest number of hospitalizations for fractures of the skull and facial bones (Figure 4).

## 4. Discussion

In the period analyzed in this study (2015–2024), Brazil exhibited a steady increase in hospitalizations due to bone fractures. This upward trend parallels the global patterns reported by the Global Burden of Disease Study 2019, which estimated 178 million new fractures worldwide in 2019, a 33.4% increase since 1990, along with 455 million prevalent cases and 25.8 million years lived with disability (YLDs) [14]. The estimated annual percent change (APC) further corroborates this sustained rise in fracture-related hospitalizations in Brazil. Brazil mirrors the global scenario, with a progressive rise in absolute fracture burden despite potential stabilization in age-standardized rates. In both contexts, the rise in absolute numbers can be related to the population aging and growth [15].

It is important to note that the global increase (33.4%) reported by the *GBD 2019* [14] represents a cumulative growth between 1990 and 2019, whereas the Brazilian APC reflects an average yearly increase between 2015 and 2024. Although they describe different time scales and statistical metrics, both indicate a consistent upward trend in fractures burden, with the Brazilian APC suggesting a more accelerated recent growth compared to the long-term global trajectory.

The most pronounced increases in femoral and other limb fractures underscore the growing impact of osteoporotic fragility fractures among older adults, compounded by injuries related to falls and traffic accidents [6,7]. As observed globally, these trends highlight the urgent need for preventive strategies targeting bone health, fall prevention, and trauma reduction, particularly in an aging population.

The analysis of sex-related fracture patterns reveals that the occurrence of fractures is not independent of sex. Men experienced nearly twice the number of hospitalizations between 2014 and 2024 compared to women. This is supported by a study on patients in Pernambuco, where a 2:1 ratio was observed between hospitalizations for femur fractures and for fractures of other limb bones in men compared with women, reinforcing the vulnerability of this group [10]. Furthermore, this disparity is widely documented in the literature, which consistently reports a higher incidence of traumatic and high-energy fractures in males due to greater exposure to motor-vehicle collisions, occupational hazards, and risk-taking behaviors [16,17].

Sex is strongly associated with fracture patterns at the population level. The analysis of hospitalizations between men and women by fracture type revealed a marked epidemiological divergence in fracture-related hospitalizations, a pattern widely documented in the literature [18,19]. Among young and middle-aged men (20–59 years), the high burden of fractures of other limb bones, along with the predominance of skull, facial, and thoracic/pelvic injuries, is consistent with high-energy trauma mechanisms [20,21]. In contrast, the pattern observed in women is substantially different; hospitalizations rise only at older ages, particularly among those aged 80 years or older, with femur fractures becoming the dominant injury type.

The literature supports this finding, reporting a higher prevalence of proximal femur fractures in women and highlighting the biomechanical vulnerability associated with postmenopausal osteoporosis, which predisposes older women to low-energy fractures, most commonly resulting from ground-level falls increases and contributes to fracture risk [8,9,15]. This age concentration is particularly relevant, as femoral fractures occurring at very advanced ages (≥80 years) are strongly associated with frailty, sarcopenia, and comorbidity burden, well-established predictors of postoperative complications and mortality [6,8]. Accordingly, although women present lower overall hospitalization rates compared to men, the higher concentration of femur fractures at advanced ages likely contributes to the elevated case-fatality observed in this group.

At the population level, fracture patterns among men are more frequently associated with high-energy trauma, underscoring the need for targeted injury-prevention and public-health strategies that account not only for sex differences but also for life-course stage and the predominant mechanisms of injury within each group.

The analysis of years of life lost (YLL) further reinforces the divergent mechanisms underlying fracture-related outcomes in men and women. Femur fractures accounted for a large proportion of total YLL and yet were characterized by relatively low mean YLL per death, reflecting mortality concentrated later in life, which is corroborated with number of hospitalizations by femur fracture in the 80 year and over [22,23]. Interestingly, Silva et al. (2024) [12] showed that male sex and age are risk factors for increased one-year mortality following femur surgery. This higher mortality has been attributed to multifactorial causes, including smoking and alcohol consumption, combined with severe comorbidities, particularly pulmonary and cardiovascular diseases, which accounted for 36.4% and 18.2% of deaths, respectively [12].

Across all fracture categories, males consistently exhibited higher mean YLL per death, indicating a greater burden of premature mortality. Although femur fractures accounted for the largest share of total YLL due to their high frequency and lethality in older adults, fracture types more prevalent among younger men, particularly skull/facial and thoracic/pelvic injuries, contributed proportionally less to overall YLL but displayed markedly elevated mean YLL values. This pattern suggests that high-energy trauma mechanisms are associated with substantial premature mortality, especially among men [14,24]. The comparison of mean YLL across sexes showed that males consistently experienced greater years of life lost for every fracture category, aligning with the higher lethality of early-life traumatic injuries in this group. Taken together, these findings confirm that while women bear the burden of fracture-related mortality at advanced ages due to skeletal fragility, at the population level, premature mortality is more frequently associated with high-impact trauma among men [25]. These sex-specific trajectories highlight the need for complementary public-health approaches, including traffic and occupational safety policies broadly targeting men, alongside osteoporosis management and fall-prevention strategies directed toward older women [26].

In our dataset, the Northeast region included states with some of the lowest numbers of total fracture hospitalizations, such as Amazonas, Acre, Roraima, and Amapá, however also included a state with the highest count of hospitalization, which was Rondônia. This pattern reflects the overall distribution of all fracture types, which encompasses a wide range of injuries with different mechanisms, severities, and healthcare pathways. In contrast to this data, in a study conducted by Modesto et al. (2022), covering the period from 2008 to 2021 and focusing exclusively on femoral fractures, the region with the highest number of hospitalizations was the Southeast, followed by the Northeast, with males being the most frequently hospitalized sex [27].

Our analysis also pointed out Piauí as one of the states with a higher number of hospitalizations. Peterle et al. (2020) reported that Piauí registered 6,480,652 hospitalizations for femur fractures in the population over 60 years old in Brazil, between 2008 and 2018, most of which occurred in women aged 80 years or older [8], similar to our findings about the gender profile of femur-related hospitalizations. However, in contrast to our data, Soares reported that the higher total number of hospitalizations, between 2011 and 2016, occurred in the Southeast region, with this region also showing the highest occurrence of femoral fracture-related admissions in both females and males [28].

It is important to note that studies frequently focus on femoral fractures because they represent one of the most clinically severe and resource-intensive types of musculoskeletal injury. Hip and femur fractures are strongly associated with high mortality, long hospital stays, the need for surgical intervention, and substantial costs to the health system [8,29]. However, this femur-specific focus introduces important limitations. While femur fractures provide valuable information about severe orthopedic injuries and elderly health, they offer only a partial view of the national fracture profile, meaning that broader and more comprehensive datasets are still needed to fully understand the epidemiology of fractures across different age groups, mechanisms of injury, and regions.

With respect to the ethnic distribution of hospitalized patients, findings from previous studies contrast with the results of the present analysis. In a study by Siqueira et al. (2005), White patients accounted for the largest share of hospitalizations, followed by Black patients and then individuals categorized as mixed-race [30]. Previous studies report differences in average peak bone mass between population groups, with some studies indicating higher mean values among Black populations, potentially contributing to lower osteoporosis prevalence at the population level [31,32].

When compared with other groups, Indigenous individuals were the most affected by fractures involving multiple body regions and by injuries classified as “other body regions”. Black and brown individuals showed a higher occurrence of skull and facial fractures, whereas white individuals were more frequently affected by femoral fractures. The association between skull and facial fractures and Black and brown populations reflects socioeconomic rather than biological determinants. These injuries, typically resulting from motorcycle accidents or interpersonal violence, are linked to high-energy trauma [11,33].

According to the 2022 Census, 72.9% of residents of favelas and urban informal settlements self-identify as Black or brown [34]. Regarding Indigenous individuals, the 2022 Census reveals that their population grew by approximately 89% between 2010 and 2022, and that over half (about 54%) of Indigenous people now live in urban areas, including outside Indigenous territories [34]. These figures suggest that the higher occurrence of multiple fractures and fractures in other body regions among Black/brown and Indigenous populations may reflect population-level structural and socioeconomic inequalities.

## 5. Limitations

This study has some limitations. First, its ecological and population-based design precludes individual-level inference. Associations observed between sex, ethnicity, region, and fracture patterns reflect aggregated distributions and may be subject to ecological fallacy. Second, the analysis relied on secondary data from SIH/SUS, which may be affected by underreporting, miscoding, and regional heterogeneity in data quality. In addition, the database includes only hospitalizations within the public healthcare system (SUS), excluding cases managed exclusively in the private sector. Third, information on the mechanism of injury and individual clinical variables (e.g., comorbidities, frailty, time to surgery) was not available, limiting etiological interpretation. Fourth, hospitalization rates were presented as crude rates per 100,000 inhabitants. Although age-stratified analyses were performed, formal age-standardized rates were not calculated, and temporal or regional comparisons may therefore be partially influenced by demographic aging. Finally, YLL estimates were derived from aggregated age categories using representative midpoint ages, which may introduce approximation bias, particularly in the open-ended ≥80 age group. Despite these constraints, the nationwide scope and multidimensional stratification provide a comprehensive overview of fracture burden in Brazil.

## 6. Conclusions

This nationwide analysis reveals a sustained increase in fracture-related hospitalizations in Brazil, alongside marked disparities across sex, age, fracture type, region, and ethnicity, underscoring the complex and multifactorial nature of fracture burden in the country. Men were disproportionately affected by high-energy trauma, resulting in greater premature mortality, while women experienced a sharp concentration of femur fractures at older ages, reflecting the growing influence of population aging and skeletal fragility. Regional and ethnic inequalities further highlighted social and structural vulnerabilities that shape exposure to trauma and access to care. By providing a comprehensive and disaggregated overview of national trends, this study offers critical evidence to guide the development of targeted public-health policies, ranging from traffic and occupational injury prevention to fall-prevention and osteoporosis management, and to strengthen trauma care systems in the most vulnerable populations. Such epidemiological monitoring is essential for informed decision-making, resource allocation, and the design of effective strategies aimed at reducing fractures and improving outcomes across Brazil.

## Figures and Tables

**Table 1:** Annual Percent Change (APC) in Fracture-Related Hospitalizations, 2014–2024.

Fracture Type	APC	95% CI	*p*-Value
Femur fracture	4.31	3.76–4.85%	*p* < 0.001
Fracture of the neck, thorax, or pelvis	4.47	3.58–5.36%	*p* < 0.001
Fracture of the skull and facial bones	1.61	–0.28–3.52	*p* = 0.134
Fractures of other limb bones	4.51	3.65–5.39%	*p* < 0.001
Multiple region/body fractures	6.13	5.29–6.97%	*p* < 0.001

CI: Confidence Interval.

**Table 2:** Sex-specific Hospitalization Rates, Mortality, and Odds Ratios for Different Fracture Types in Brazil, 2015–2024.

	Hospitalizations		Hospitalizations per Fracture (%)		Odds Ratio	95% CI	Deaths		Fatality (%)	
Fracture Type	Female	Male	Female	Male			Female	Male	Female	Male
Femur fracture	340,944	514,981	16.5	11.3	1.55	1.54–1.56	23,379	14,587	6.86	2.83
Fracture of neck. thorax. or pelvis	56,201	154,350	2.7	3.4	0.80	0.79–0.81	1261	4051	2.24	2.62
Fracture of the skull and facial bones	56,994	256,554	2.8	5.6	0.48	0.50–0.51	292	1684	0.51	0.66
Fractures of other limb bones	1,477,107	3,336,181	71.6	73.4	0.92	0.91–0.92	2317	4633	0.16	0.14
Multiple region/body fractures	131,020	284,274	6.4	6.3	1.02	1.01–1.02	4235	4042	3.23	1.42

## Data Availability

The data used in this study are publicly available from the Hospital Information System of the Brazilian Unified Health System (SIH/SUS), provided by the Brazilian Ministry of Health through DATASUS [35] and can be accessed at http://tabnet.datasus.gov.br/cgi/deftohtm.exe?sih/cnv/nruf.def (accessed on 1 August 2025).

## Supplementary Materials

The following supporting information can be downloaded at: https://www.mdpi.com/article/10.3390/epidemiologia7030063/s1: Supplementary Data S1. Fracture-related hospitalizations stratified by fracture type, sex, and age group, Brazil, 2015–2024; Supplementary Data S2. Population-adjusted hospitalization rates for bone fractures by Brazilian state, 2015–2024.

## Abbreviations

The following abbreviations are used in this manuscript:

APC	Annual Percent Change
CFR	Case Fatality Rate
CI	Confidence Interval
COVID-19	Coronavirus Disease 2019
DATASUS	Department of Informatics of the Unified Health System (Brazil)
GBD	Global Burden of Disease
IBGE	Brazilian Institute of Geography and Statistics
ICD-10	International Classification of Diseases, 10th Revision
OR	Odds Ratio
PYLL	Potential Years of Life Lost
RR	Relative Risk
SIH/SUS	Hospital Information System of the Unified Health System
SUS	Unified Health System (Sistema Único de Saúde)
YLD	Years Lived with Disability
YLL	Years of Life Lost
